# Risk factor compositions of nonalcoholic fatty liver disease change with body mass index in males and females

**DOI:** 10.18632/oncotarget.9691

**Published:** 2016-05-30

**Authors:** Long Wang, Jinghui Guo, Jianping Lu

**Affiliations:** ^1^ Department of Gastroenterology, Shanghai Jiao Tong University Affiliated Sixth People's Hospital, Shanghai, China; ^2^ Health Examination Center, Shanghai Jiao Tong University Affiliated Sixth People's Hospital, Shanghai, China

**Keywords:** nonalcoholic fatty liver disease, body mass index, gender difference, risk factor compositions, nonalcoholic steato-hepatitis, Pathology Section

## Abstract

Nonalcoholic fatty liver disease (NAFLD) is highly prevalent and correlated with obesity. To evaluate the role of body mass index (BMI) and gender difference in NAFLD, 8817 general adult subjects underwent physical examinations and were divided into four groups: underweight, normal, overweight and obese. The risk factor compositions for NAFLD were evaluated in each group by gender. The percentage of subjects with NAFLD increased sharply from 0.4% in the underweight group up to 81.9 % in the obese group. BMI stratification showed distinct risk factor compositions associated with NAFLD in males and females according to BMI and improved the performance of NAFLD prediction models in each group. Triglycerides (TG), alanine transaminase (ALT), aspartate transaminase (AST), and uric acid were steady risk factors for NAFLD in males. Total cholesterol (TC), TG, low-density lipoprotein cholesterol (LDL-C), ALT, and uric acid were steady risk factors for NAFLD in females. TG, ALT and uric acid were common risk factors in both genders with high performance for NAFLD discrimination. Our data provide gender- and BMI-specific risk factor compositions that will facilitate individualised treatment and benefit NAFLD control and prevention.

## INTRODUCTION

Fatty liver refers to hepatic steatosis accounting for more than 5–10% of the total weight of the liver or macrosteatosis of the same extent in histopathology [1]. Excessive consumption of alcohol (females > 20 g/d, males > 30 g/d) leads to alcoholic fatty liver [2]. While nonalcoholic fatty liver disease (NAFLD) is a spectrum of hepatic disorders, ranging from simple or bland fatty liver to nonalcoholic steato-hepatitis (NASH), which is now more common than alcoholic liver disease owing to the rapid rise in the prevalence of obesity [1–3].

NAFLD was first described in 1980 and is divided into the histological categories of nonalcoholic fatty liver and NASH [4]. Up to 75% of individuals with NAFLD are patients with isolated hepatic steatosis or with steatosis concomitant of mild nonspecific inflammation; up to 25% of individuals with NAFLD also have NASH [4–7]. Stages of isolated hepatic steatosis, steatosis with mild lobular inflammation and NASH can evolve as each other and patients with NASH have a high risk of developing liver fibrosis and liver cirrhosis [1–7]. Diabetes, insulin resistance, hypertension, weight gain and increasing alanine aminotransferase (ALT) and aspartate aminotransferase (AST) are known risk factors for disease progression from NASH to liver fibrosis [4]. Although less than 4% of individuals with nonalcoholic fatty liver will progress to cirrhosis directly, up to 20% of subjects with NASH are expected to progress to liver cirrhosis [4]. Thus the impact of NAFLD on liver disease is receiving increasing attention.

NAFLD, the hepatic manifestation of the metabolic syndrome, is the most common liver disease worldwide with prevalence estimates ranging from 25% to 45% [8, 9]. The proportion of NAFLD among chronic liver diseases rose from 47% to 75% between 1988 and 2008 according to data from the US National Health and Nutrition Examination Survey [10]. It is worth noting that the known risk factors for NASH—obesity, visceral obesity, type 2 diabetes, insulin resistance and arterial hypertension—are increasing in parallel with that of NAFLD from 21.74% to 33.22%, 35.18% to 51.43%, 5.55% to 9.11%, 23.29% to 35.00% and 22.68% to 34.08%, respectively [11]. These risk factors together with NAFLD not only constitute the leading protagonists of metabolic diseases but also have reciprocal causation and act as intermediary exacerbating factors for various diseases, including cardiovascular disease [12–14].

The risk factors for NAFLD are divided into genetics, metabolic changes, hormone changes, inflammation, diet, physical activity and the gut microbiota [15]. Encouraging progress has been made in noninvasive evaluation of NAFLD, including steatosis grading, NASH diagnosis, and liver fibrosis staging [16–22]. Studies on risk factor have resulted in the formulation of NAFLD prediction algorithms, which include various parameters such as BMI, TC, TG, glucose, age, gender, waist circumference, γ-glutamyltranspeptidase, metabolic syndrome, type 2 diabetes, fasting insulin level, fasting AST level, and the AST/ALT ratio [16–20]. However, these prediction strategies have not significantly improved the clinical diagnosis of NAFLD, because there is no evidence that the performance of these algorithms exceeds that of the clinical, laboratory, and imaging evaluations that are routinely performed in patients with suspected NAFLD [21]. Thus, improvement of NAFLD prediction remains essential. Obesity is a major risk factor for NAFLD; indeed, obesity shares many risk factors with NAFLD [22]. Therefore, the confounding factors of obesity and NAFLD must be controlled for to identify NAFLD-specific risk factors. Unfortunately, of the above representative prediction algorithms [16–20], only four of five studies included obesity indexes. However, in studies that evaluated BMI and/or waist circumference [16–20], no stratification of obesity was performed to distinguish the impact of obesity on NAFLD and its risk factors. In addition, age, gender, and ethnicity are associated with the prevalence of NAFLD [9], but only one [16] and two [16,18] of the five above studies included age and gender as variables in their predictive equations. Thus, much important information might be lost when using these algorithms. Recent studies have focused mainly on NASH instead of NAFLD [4,6], and the incidence of NAFLD has been investigated in only one study [9]. Therefore, further studies are needed to assess the incidence of NAFLD according to age, ethnicity, and geographic location.

In this study, to evaluate the role of BMI and gender in NAFLD, 8817 general Chinese subjects living in Shanghai were divided into underweight, normal weight, overweight and obese groups. The patterns of association between age, hypertension, fasting glucose, serum lipid profile, serum metabolic parameters, and NAFLD were evaluated and compared in each group according to gender.

## RESULTS

### The distribution tendency of parameters in groups classified by BMI

In total, 8817 subjects were enrolled in this analysis. The numbers of subjects in the underweight (BMI < 18.5), normal (18.5 ≤ BMI ≤ 23.9), overweight (24.0 ≤ BMI ≤ 27.9) and obese (BMI ≥ 28.0) groups were 445 (5.0%), 4899 (55.6%), 2801 (31.8%) and 672 (7.6%), respectively (Table 1). The percentages of subjects with NAFLD increased from 0.4% in the underweight group to 82.4% in the obese group. The percentages of males and females with NAFLD also increased sharply with increasing BMI. There was no significant difference in the percentages of subjects with NAFLD between males and females in the underweight, overweight and obese groups. However, the percentage of males with NAFLD was significantly higher than that of females in the normal body weight group (14.4% vs. 11.9%, *p* = 0.0156). The BMI, age, male ratio, systolic pressure, diastolic pressure, serum levels of TC, TG, LDL-C, glucose, ALT, AST, urea nitrogen, uric acid and creatinine were higher in groups with higher BMI. In contrast, the serum HDL-C level declined gradually with increasing BMI.

**Table 1:** The distribution tendency of parameters in groups classified by BMI

Indexes	BMI < 18.5	18.5 < BMI < 23.9	24.0 < BMI < 27.9	BMI > 28.0	*P*
	(*N* = 445, 5.0%)	(*N* = 4899, 55.6%)	(*N* = 2801, 31.8%)	(*N* = 672, 7.6%)	
BMI (kg/m^2^)	17.8 (17.2–18.2)	21.6 (20.2–22.8)	25.5 (24.7–26.4)	29.3 (28.5–30.6)	*<0.001*
Age, yrs	32 (26–48)	43 (32–58)	51 (38–61)	51 (37–63)	*<0.001*
Males, N (%)	72 (16.2%)	1628 (33.2%)	1699 (60.7%)	435 (64.7%)	*<0.001*
Systolic pressure (mmHg)	117.1 + 15.9	124.5 + 17.4	134.4 + 17.9	143.0 + 19.3	*<0.001*
Diastolic pressure (mmHg)	70.5 + 9.6	75.5 + 11.0	81.8 + 11.2	86.3 + 11.7	*<0.001*
TC (mM)	4.6 + 0.9	4.8 + 0.9	5.1 + 1.0	5.2 + 1.0	*<0.001*
TG (mM)	0.8 + 0.3	1.11 + 0.87	1.66 + 1.35	2.0 + 1.5	*<0.001*
HDL-C (mM)	1.8 + 0.4	1.6 + 0.4	1.39 + 0.33	1.30 + 0.32	*<0.001*
LDL-C (mM)	2.5 + 0.7	2.8 + 0.8	3.12 + 0.81	3.25 + 0.80	*<0.001*
Glucose (mM)	5.0 + 1.2	5.2 + 1.1	5.6 + 1.3	5.8 + 1.6	*<0.001*
ALT (U/L)	13.4 + 8.3	16.1 + 12.3	23.4 + 18.1	31.9 + 29.4	*<0.001*
AST (U/L)	19.8 + 5.9	20.3 + 8.0	22.9 + 10.8	25.8 + 13.4	*<0.001*
Urea nitrogen (mM)	4.6 + 1.4	4.8 + 1.2	5.1 + 1.3	5.2 + 1.4	*<0.001*
Uric acid (μM)	252.7 + 61.5	284.8 + 69.9	336.7 + 75.9	370.1 + 82.2	*<0.001*
Creatinine (μM)	58.6 + 13.0	62.7 + 14.9	69.8 + 16.0	71.3 + 17.2	*<0.001*
NAFLD, N (%)	2 (0.4%)	624 (12.7%)	1379 (49.2%)	554 (82.4%)	*<0.001*
Males with NAFLD, N (%)	0 (0.0%)	234 (14.4%)^*^	830 (48.9%)	360 (82.8%)	*<0.001*
Females with NAFLD, N (%)	2 (0.5%)	390 (11.9%)	549 (49.8%)	194 (81.9%)	*<0.001*

Normally distributed data are presented as the means ± standard deviation (SD); skewed data are presented as the medians (interquartile range); and categorical data are presented as the numbers (percentage). Differences among groups were examined using Kruskal-Wallis H test, one-way ANOVA, χ2 tests according to the data distribution tendency. Abbreviations: BMI, body mass index; TC, total cholesterol; TG, triglycerides; HDL-C, high-density lipoprotein cholesterol; LDL-C, low-density lipoprotein cholesterol; ALT, alanine transaminase; AST, glutamic-oxalacetic transaminase; NAFL, nonalcoholic fatty liver. According to the categories recommended by the Chinese government, BMI < 18.5 refers to underweight; 18.5 < BMI < 23.9 refers to healthy weight; 24.0 < BMI < 27.9 refers to overweight; BMI > 28.0 refers to obese.

* *P* = 0.0156.

### Risk factors associated with NAFLD without BMI stratification

To learn the risk factors associated with NAFLD in multiple logistic regression analysis ignoring BMI stratification, the general population was categorised by gender. The male and female groups comprised 3834 (43.5%) and 4983 (56.5%) subjects, respectively. For binary logistic regression analysis, NAFLD was used as the dependent variable, whereas BMI, age, systolic pressure, diastolic pressure, TC, TG, HDL-C, LDL-C, glucose, ALT, AST, urea nitrogen, uric acid, and creatinine were used as independent variables. All continuous variables were introduced into the starting model using actual values. As shown in Table 2, BMI, age, diastolic pressure, TG, LDL-C, glucose, ALT, AST, uric acid and creatinine are independent risk factors for NAFLD in both males and females. TC is an independent risk factor for females but not males (Table 2). Systolic pressure displayed a tendency to be an independent risk factor for males. HDL-C and urea nitrogen were not independent risk factors for either males or females.

Almost all existing reports had ignored the BMI stratification, their analytical logic was similar to what we done above. Because NAFLD was rarely observed in subjects with BMI < 18.5 and the proportion of subjects with NAFLD increased sharply from the underweight group to the obese group, we then stratified the population into normal body weight, overweight, and obese groups to assess the impact of BMI on NAFLD and its risk factors.

**Table 2:** Risk factors associated with NAFLD in multiple logistic regression analysis ignoring BMI stratification

Risk factor	Males (*N* = 3834, 43.5%)		Females (*N* = 4983, 56.5%)	
	OR (95% CI)	*P*	OR (95% CI)	*P*
BMI (kg/m^2^)	1.44 (1.38 ~ 1.50)	*<0.001*	1.42 (1.37 ~ 1.48)	*<0.001*
Age, yrs	1.01 (1.00 ~ 1.02)	*0.003*	1.03 (1.02 ~ 1.040	*<0.001*
Systolic pressure (mmHg)	0.99 (0.99 ~ 1.00)	*0.059*	/	*/*
Diastolic pressure (mmHg)	1.02 (1.01 ~ 1.03)	*0.001*	1.02 (1.01 ~ 1.03)	*0.002*
TC (mM)	/	*/*	0.40 (0.32 ~ 0.52)	*<0.001*
TG (mM)	1.16 (1.08 ~ 1.25)	*<0.001*	2.01 (1.75 ~ 2.29)	*<0.001*
HDL-C (mM)	/	*/*	/	*/*
LDL-C (mM)	1.38 (1.23 ~ 1.55)	*<0.001*	3.14 (2.34 ~ 4.22)	*<0.001*
Glucose (mM)	1.18 (1.11 ~ 1.26)	*<0.001*	1.17 (1.08 ~ 1.27)	*<0.001*
ALT (U/L)	1.06 (1.05 ~ 1.07)	*<0.001*	1.06 (1.04 ~ 1.07)	*<0.001*
AST (U/L)	0.95 (0.94 ~ 0.97)	*<0.001*	0.95 (0.93 ~ 0.97)	*<0.001*
Usea nitrogen (mM)	/	*/*	/	*/*
Uric acid (μM)	1.01 (1.00 ~ 1.01)	*<0.001*	1.01 (1.00 ~ 1.01)	*<0.001*
Creatinine (μM)	0.99 (0.98 ~ 0.99)	*<0.001*	0.98 (0.97 ~ 0.99)	*<0.001*

OR, odds ratio; CI, confidence interval. For abbreviations, see Table 1. ORs for continuous variables = OR for an increase of 1 unit.

### Risk factors associated with NAFLD in subjects with normal body weight

To identify the risk factors associated with NAFLD in those with a normal body weight (18.5 < BMI < 23.9), a binary logistic regression analysis was performed. Age and the serum levels of TG, LDL-C, glucose, ALT and uric acid were found to be positively associated with NAFLD in both males and females (Table 3). Serum levels of TC and AST were negatively associated with NAFLD in both males and females (Table 3). Although the percentage of females with NAFLD was significantly lower than that of males with a normal body weight (Table 1), three further risk factors—BMI, diastolic pressure, and serum creatinine level—were independent risk factors for NAFLD only in females (Table 3). Systolic blood pressure, HDL-C, and urea nitrogen levels were not independent risk factors for NAFLD in males or females (Table 3).

**Table 3:** Risk factors associated with NAFLD in multiple logistic regression analysis in subjects with 18.5 < BMI < 23.9

Risk factor	Males (*N* = 1628)		Females (*N* = 3271)	
	OR (95% CI)	*P*	OR (95% CI)	*P*
BMI (kg/m^2^)	/	/	1.47 (1.33 – 1.63)	<0.001
Age, yrs	1.02 (1.01 – 1.030	<0.001	1.04 (1.03 – 1.05)	<0.001
Systolic pressure (mmHg)	/	/	/	/
Diastolic pressure (mmHg)	/	/	1.02 (1.01 – 1.04)	<0.001
TC (mM)	0.42 (0.26 – 0.70)	0.001	0.48 (0.33 – 0.69)	<0.001
TG (mM)	1.58 (1.37 – 1.82)	<0.001	1.96 (1.64 – 2.35)	<0.001
HDL-C (mM)	/	/	/	/
LDL-C (mM)	3.45 (1.99 – 5.98)	<0.001	2.87 (1.88 – 4.39)	<0.001
Glucose (mM)	1.18 (1.05 – 1.32)	0.007	1.18 (1.07 – 1.31)	0.001
ALT (U/L)	1.06 (1.04 – 1.08)	<0.001	1.06 (1.04 – 1.08)	<0.001
AST (U/L)	0.94 (0.90 – 0.98)	0.001	0.93 (0.90 – 0.96)	<0.001
Usea nitrogen (mM)	/	/	/	/
Uric acid (μM)	1.01 (1.00 – 1.01)	<0.001	1.01 (1.00 – 1.01)	<0.001
Creatinine (μM)	/	/	0.98 (0.96 – 0.99)	0.009

OR, odds ratio; CI, confidence interval. For abbreviations, see Table 1. ORs for continuous variables = OR for an increase of 1 unit.

Compared to the above analysis without BMI stratification (Table 2), in subjects with normal body weight, age, TG, LDL-C, glucose, ALT, AST, and uric acid were consistent risk factors in both males and females. BMI, diastolic blood pressure, and serum creatinine levels were independent risk factors for NAFLD only in females; systolic pressure was a risk factor for neither males nor females. Taken together, our data suggest that BMI stratification changes the NAFLD risk factor compositions for both males and females.

### Risk factors associated with NAFLD in overweight subjects

In overweight subjects, BMI and levels of TG, LDL-C, glucose, ALT, AST, uric acid, and creatinine were independent risk factors for both males and females; age and TC were female-specific risk factors (Table 4). Compared to subjects with normal body weight, BMI, diastolic pressure and levels of HDL-C and creatinine emerged as independent risk factors for males; age and TC level lost the independent association with NAFLD in males. The risk factor panel of females was relatively stable, with the exception of diastolic pressure, which lost the independent association with NAFLD. Compared to the above analysis without BMI stratification (Table 2), in overweight subjects, BMI, TG, LDL-C, glucose, ALT, AST, uric acid, and creatinine were consistent risk factors in both males and females. The serum TC level was a consistent risk factor negatively associated with NAFLD only in females. Age and diastolic pressure were risk factors only for females and males, respectively. Similar to subjects with a normal body weight, systolic pressure was a risk factor for neither males nor females. Therefore, our data verify that BMI stratification affects the NAFLD risk factor compositions.

**Table 4:** Risk factors associated with NAFLD in multiple logistic regression analysis in subjects with 24.0 < BMI < 27.9

Risk factor	Males (*N* = 1699)		Females (*N* = 1102)	
	OR (95% CI)	*P*	OR (95% CI)	*P*
BMI (kg/m^2^)	1.38 (1.24 – 1.53)	<0.001	1.47 (1.29 – 1.68)	<0.001
Age, yrs	/	/	1.03 (1.01 – 1.04)	<0.001
Systolic pressure (mmHg)	/	/	/	/
Diastolic pressure (mmHg)	1.02 (1.00 – 1.03)	0.007	/	/
TC (mM)	/	/	0.36 (0.25 – 0.53)	<0.001
TG (mM)	1.10 (1.01 – 1.20)	0.035	1.91 (1.55 – 2.36)	<0.001
HDL-C (mM)	0.49 (0.32 – 0.78)	0.002	/	/
LDL-C (mM)	1.33 (1.14 – 1.55)	<0.001	3.09 (1.96 – 4.86)	<0.001
Glucose (mM)	1.20 (1.10 – 1.31)	<0.001	1.16 (1.01 – 1.34)	0.042
ALT (U/L)	1.05 (1.04 – 1.07)	<0.001	1.06 (1.04 – 1.09)	<0.001
AST (U/L)	0.96 (0.94 – 0.98)	<0.001	0.96 (0.93 – 0.99)	0.006
Urea nitrogen (mM)	/	/	/	/
Uric acid (μM)	1.01 (1.00 – 1.01)	<0.001	1.01 (1.00 – 1.01)	<0.001
Creatinine (μM)	0.99 (0.98 – 0.99)	<0.001	0.96 (0.95 – 0.98)	<0.001

OR, odds ratio; CI, confidence interval. For abbreviations, see Table 1. ORs for continuous variables = OR for an increase of 1 unit.

### Risk factors associated with NAFLD in obese subjects

TG, ALT and uric acid levels were independent risk factors associated with NAFLD in both males and females with a BMI > 28.0. Compared to subjects who were overweight, the diastolic pressure and the levels of HDL-C, LDL-C and glucose were not associated with NAFLD in males (Tables 5). The BMI, age, levels of glucose, AST and creatinine were not associated with NAFLD in females, whist HDL-C levels emerged as an independent risk factor in females. Compared to the analysis without BMI stratification (Table 2), in obese subjects, only TG, ALT, and uric acid were consistent risk factors in both males and female. Serum TC level was a consistent risk factor negatively associated with NAFLD only in females. However, BMI, AST, and creatinine were risk factors only in males; age, systolic blood pressure, diastolic pressure, LDL-C, and glucose lost their independent association with NAFLD in males. Interestingly, BMI, age, systolic blood pressure, diastolic blood pressure, glucose, AST, and creatinine lost their independent association with NAFLD in females. In conclusion, BMI dramatically alters the NAFLD risk factor compositions, and risk factor compositions differ according to BMI stratification.

**Table 5:** Risk factors associated with NAFLD in multiple logistic regression analysis in subjects with BMI > 28.0

Risk factor	Males (*N* = 435)		Females (N = 237)	
	OR (95% CI)	*P*	OR (95% CI)	*P*
BMI (kg/m^2^)	1.23 (1.01 – 1.49)	0.039	/	/
Age, yrs	/	/	/	/
Systolic pressure (mmHg)	/	/	/	/
Diastolic pressure (mmHg)	/	/	/	/
TC (mM)	/	/	0.11 (0.03 – 0.45)	0.002
TG (mM)	1.58 (1.13 – 2.21)	0.007	2.83 (1.29 – 6.18)	0.009
HDL-C (mM)	/	/	7.43 (1.33 – 41.56)	0.023
LDL-C (mM)	/	/	15.16 (3.15 – 73.00)	0.001
Glucose (mM)	/	/	/	/
ALT (U/L)	1.05 (1.02 – 1.09)	0.001	1.10 (1.03 – 1.17)	0.003
AST (U/L)	0.96 (0.93 – 0.99)	0.21	/	/
Urea nitrogen (mM)	/	/	/	/
Uric acid (μM)	1.00 (1.00 – 1.01)	0.44	1.01 (1.00 – 1.01)	0.032
Creatinine (μM)	0.97 (0.95 – 0.99)	0.016	/	/

OR, odds ratio; CI, confidence interval. For abbreviations, see Table 1. ORs for continuous variables = OR for an increase of 1 unit.

### The AUCs of each NAFLD prediction model, steady risk factors and integrated steady risk factors

The above data show that the risk factor compositions in males and females are considerably affected by BMI. While the levels of TG, ALT, AST and uric acid were steady risk factors in males with any BMI; the levels of TC, TG, LDL-C, ALT and uric acid were steady risk factors in females with any BMI (Table 6). The levels of TG, ALT and uric acid were steady risk factors in both males and females with any BMI (Table 6, bold labelled). Although the diastolic blood pressure was specifically associated with healthy weight females and overweight males, our data suggest that NAFLD is the result of multiple factors, and no single factor is responsible for its pathogenesis solely. To further show the importance of BMI stratification, the performance of each NAFLD prediction model, steady risk factor and integrated steady risk factor, was calculated according to gender. Of the steady risk factors, TG showed the highest AUCs both in males (0.74) and females (0.80); the AUCs of the integrated steady risk factors for males (TG, ALT, AST, and uric acid) and females (TC, TG, LDL-C, ALT, and uric acid) were 0.79 (0.78–0.81) and 0.84 (0.83–0.85), respectively (Table 6). As expected, models specific to the various BMI stratifications showed improved AUCs. The AUCs of models for males (variables: age, TC, TG, LDL-C, glucose, ALT, AST, and uric acid) and females (variables: BMI, age, diastolic pressure, TC, TG, LDL-C, glucose, ALT, AST, uric acid, and creatinine) of a healthy weight were 0.86 (0.84–0.87) and 0.90 (0.89–0.91), respectively (Table 6). The AUCs of models for overweight males (variables: BMI, diastolic pressure, TG, HDL-C, LDL-C, glucose, ALT, AST, uric acid, and creatinine) and females (variables: BMI, age, TC, TG, LDL-C, glucose, ALT, AST, uric acid, and creatinine) were 0.86 (0.85–0.87) and 0.90 (0.89–0.91), respectively (Table 6). The AUCs declined in obese subjects, those for males (variables: BMI, TG, ALT, AST, uric acid, and creatinine) and females (variables: TC, TG, HDL-C, LDL-C, ALT, and uric acid) being 0.85 (0.83–0.86) and 0.81 (0.79–0.82), respectively (Table 6). The cut-off values of each prediction equation for male and female subjects who were healthy weight, overweight, or obese were 0.151 (sensitivity: 0.821, specificity: 0.817) and 0.126 (sensitivity: 0.856, specificity: 0.843), 0.472 (sensitivity: 0.816, specificity: 0.812) and 0.466 (sensitivity: 0.842, specificity: 0.837), and 0.798 (sensitivity: 0.807, specificity: 0.802) and 0.807 (sensitivity: 0.808, specificity: 0.801), respectively. In conclusion, BMI stratification improved the performance of the NAFLD prediction models.

**Table 6:** The AUCs of each NAFLD prediction model, steady risk factors and integrated steady risk factors

Model / Risk factor	Males	*P*	Females	*P*
Models for subjects with 18.5 < BMI < 23.9				
Risk factors listed in table 3	0.86 (0.84 ~ 0.87)	<0.001	0.90 (0.89 ~ 0.91)	<0.001
Models for subjects with 24.0 < BMI < 27.9				
Risk factors listed in table 4	0.86 (0.85 ~ 0.87)	<0.001	0.90 (0.89 ~ 0.91)	<0.001
Models for subjects with BMI > 28.0				
Risk factors listed in table 5	0.85 (0.83 ~ 0.86)	<0.001	0.81 (0.79 ~ 0.82)	<0.001
The AUCs of each steady risk factor				
TC	/	/	0.65 (0.59 ~ 0.63)	<0.001
TG^*^	0.74 (0.72 ~ 0.76)	<0.001	0.80 (0.78 ~ 0.81)	<0.001
LDL-C	/	/	0.70 (0.68 ~ 0.71)	<0.001
ALT^*^	0.73 (0.72 ~ 0.75)	<0.001	0.73 (0.71 ~ 0.75)	<0.001
AST	0.61 (0.59 ~ 0.63)	<0.001	/	/
Uric acid^*^	0.65 (0.64 ~ 0.67)	<0.001	0.71 (0.69 ~ 0.72)	<0.001
Integrated performance of steady risk factors				
Steady risk factors	0.79 (0.78 ~ 0.81)	<0.001	0.84 (0.83 ~ 0.85)	<0.001

AUC, area under the receiver operating characteristics (ROC) curve; for more abbreviations, see Table 1. Data were presented as AUCs (95%CI). The predictors in each BMI stratification are shown in Table 3 - 5.

* Bold indicates common steady risk factors for both males and females.

## DISCUSSION

Together with the advancements in modern biomedical technologies and the awareness of the harm caused by NAFLD, research has progressed from risk factors to exploring a strategy to predict NAFLD and NASH [4, 23]. Much effort has focused on identifying biomarkers to predict NASH or NAFLD; however, these strategies have rarely benefited clinical practice in terms of NAFLD diagnosis or discrimination of the pathological development of NAFLD [23]. The performance of current noninvasive evaluations of NAFLD varies among patients, aetiologies, and disease stages [23]. Ultrasonography is a simple and effective tool for steatosis screening, and liver biopsy is the gold standard for diagnosis of NAFLD in clinical practice [21,26].

Many factors have been reported to be associated with NAFLD or NAFLD-related pathological processes, such as inflammation and NASH, including tumour necrosis factor-α [23], interleukin-6 [24], linoleic acid [25], miR-122 [26], miR-34a [26], and haptoglobin, apolipoprotein A1, tissue inhibitor of metalloproteinase 1 and chitinase-3-like protein 1 [21, 23]. However, these factors represented only the general pathophysiological processes of inflammation, repair, remodelling and fibrosis. Distinguishing the pathophysiological processes of NAFLD from general processes is problematic [23]. In addition, other biomarkers/panels for NASH have been described [23]. Age, sex, insulin resistance, systemic hypertension, TG, ALT, AST, GGT, AST/ALT ratio, α2-macroglobulin, haptoglobin, apolipoprotein A1, total bilirubin, fasting blood glucose and fibrosis-4 were found to be associated with NASH and have been used in various diagnostic panels [23, 27, 28]. These studies advanced our knowledge of NAFLD and NASH and guided subsequent research. However, almost all of these studies were performed as shown in our Table 2, and did not take into consideration the possibility of altered risk factor compositions according to BMI; this might reduce the performance of diagnostic panels for discrimination of NAFLD. To evaluate our hypothesis, we recruited 8817 subjects. The subjects were divided into underweight, normal weight, overweight and obese groups, and the risk factor compositions and their association patterns were evaluated according to gender. First, in subjects with BMI < 18.5 kg/m^2^, only two NAFLD patients were found among 445 subjects. The percentage of NAFLD increased sharply from 0.4% in this group to 81.9% in the obese group, indicating that BMI is associated tightly with morbidity and indicated the necessity of BMI stratification in the following analysis. Second, the NAFLD risk factor composition are affected markedly by the BMI of the subjects in both males and females, which suggests a pathophysiological dynamic characteristic of the process of NAFLD development and emphasises the importance of BMI stratification in NAFLD studies. Third, TG, ALT, AST and uric acid are steady risk factors for NAFLD in males; and TC, TG, LDL-C, ALT and uric acid are steady risk factors for NAFLD in females; these risk factors displayed high performance for discrimination of NAFLD. Fourth, TG, ALT and uric acid are the common risk factors in both males and females with high performance in discrimination of NAFLD. Fifth, although BMI was used to group the subjects, it remained a risk factor for NAFLD in two-thirds of the stratifications (the normal weight, overweight and obese groups) in both males and females, with the highest AUC value. Sixth, urea nitrogen was not a risk factor in males or females, regardless of BMI stratification. Finally, the BMI, age, male ratio, systolic pressure, diastolic pressure, serum levels of TC, TG, LDL-C, glucose, ALT, AST, urea nitrogen, uric acid and creatinine were higher in groups with higher BMI, whereas HDL-C levels decreased with BMI.

The liver is the principle location of amino acid synthesis, protein degradation, carbohydrate metabolism, cholesterol synthesis, the production of triglycerides and the bulk of lipoprotein synthesis, as well as several other regulatory and growth factors [29–31]. Thus it is understandable that liver diseases correlate with metabolic disorders [31]. Regarding the liver lipid synthesis indexes, TG exhibited the greatest increase, from 0.8 ± 0.35 mM in the underweight group to 2.0 ± 1.5 mM in obese group, and was a steady risk factor for NAFLD in both males and females. By contrast, the elevating rate of TC was relatively slower and TC was a steady risk factor for NAFLD in females only. The above evidence suggests different roles for TG and TC in the pathogenesis of NAFLD in males and females. Uric acid and creatinine are indexes of metabolic and kidney function. They increase with increasing BMI, and intriguingly, are also risk factors for NAFLD. Hyperuricemia is one of the most common metabolic disorders in clinical practice, a large number of cross-sectional and several prospective studies show that hyperuricemia is associated with increased prevalence, incidence and disease severity of NAFLD [32–34]. In this report, uric acid is a common risk factor for both males and females, which consistent with previous reports [32–34]. The serum glucose level was also higher in subjects with higher BMI, although the mean did not exceed the upper limit of normal. Serum glucose level is an independent risk factor for NAFLD in both males and females who are normal weight or overweight; interestingly, serum glucose is not a risk factor for NAFLD in males and females with obesity. Systolic and diastolic blood pressures increased gradually with increasing BMI, and the average systolic pressure exceeded the upper limit of normal in the overweight and obese groups. Furthermore, systolic and diastolic pressures were independent risk factors for NAFLD in males in certain BMI stratifications. Intriguingly, systolic pressure was not a risk factor for NAFLD in females, but diastolic pressure was a risk factor for NAFLD in females with normal body weight. Our data suggest that the pattern of association between blood pressure and NAFLD varies according to gender and BMI and between systolic and diastolic pressure.

Levels of ALT and AST, indicators of liver injury and repair [19–21], increased with increasing BMI. Although the elevated ALT and AST levels did not exceed the normal ranges in our population, ALT was a common risk factor for NAFLD in both males and females regardless of BMI stratification; AST was a steady risk factor for NAFLD in males regardless of BMI stratification; AST was also an independent risk factor for NAFLD in females, with the exception of obese females. The above findings suggest that steatosis and/or mild inflammation of NAFLD lead to subclinical hepatocytes damage, which characterized by the stable association between elevated ALT and AST levels and NAFLD. It has been considered that AST/ALT > 0.8 indicates NASH and AST/ALT > 1.0 indicates progressive hepatic fibrosis [21,35]. Intriguingly, the overall average AST/ALT ratios (without gender stratification) were >1.0 and increased from 1.0 ± 0.4 in the obese group to 1.7 ± 0.6 in the underweight group. Again, we emphasise that our subjects were recruited from the general adult population and underwent physical examinations; individuals with significant alcohol consumption (>20 g per day), medications, parenteral nutrition, Wilson's disease, severe malnutrition, haemochromatosis, autoimmune liver disease, and/or chronic hepatitis were excluded. The AST and ALT levels of all participants were in the normal ranges. These factors might explain the >1.0 average AST/ALT ratios in each group. Because existing noninvasive NASH evaluation strategies using the AST/ALT ratio are derived mainly from patients with chronic hepatitis and have not been validated in different ethnicities or at multiple centres [23], we then had given up the analysis of the risk factors for AST/ALT ≥ 1.0 or 0.8. Thus, although we could not distinguish NASH subjects from NAFLD population pathologically, the normal liver function and the exclusion criteria of participants made the subjects involved were mainly NAFLD but not NASH. Because NASH develops from NAFLD and weight loss is important for all patients with NAFLD [4], control and prevention of NAFLD is a cost-effective measure to reduce the morbidity of NASH. Our findings indicate a gender- and BMI-specific risk factor composition, which will facilitate individualised treatment strategies.

The limitations of this report are: 1) we did not include all known risk factors for NAFLD and hence we could also not provide a comprehensive assessment of the correlations between risk factors and NAFLD; 2) we did not categorise the pathological stages of NAFLD, so the associations of these factors with inflammation and NASH are unknown; 3) we did not perform liver biopsy, therefore our NAFLD diagnosis was based on a noninvasive approach.

## MATERIALS AND METHODS

### Participants

The study was approved by the Review Board of Shanghai Jiao Tong University Affiliated Sixth People's Hospital and conducted according to the principles of the World Medical Association Declaration of Helsinki. All subjects provided written informed consent prior to participating in this study. The participants were simultaneously informed of their right to repeal consent by them or their kin, caretakers, or guardians.

To investigate the risk factors associated with NAFLD in the general adult population, subjects that underwent routine physical examination in our hospital were recruited sequentially from June 2014 to August 2014. All participants underwent physical examinations and anthropometry, blood biochemistry and abdominal ultrasound evaluations. The diagnosis of NAFLD was established by following the Hepatic Steatosis Ultrasound Images Assessment Procedures Manual [36]. Briefly, the following ultrasonographic features were assessed, scored, and recorded: liver to kidney contrast; parenchymal brightness (i.e., hyperechogenic liver tissue with fine, tightly packed echoes); deep beam attenuation (i.e., the decreased ability of the ultrasound beam to penetrate the liver tissue causing posterior darkness and loss of definition of the diaphragm); bright vessel walls (i.e., the presence of bright walls of small intrahepatic vessels including the portal or hepatic veins); and gallbladder wall definition. The final diagnosis was based on the presence or absence of these five criteria. Individuals with significant alcohol consumption (>20g per day), medications, parenteral nutrition, Wilson's disease, severe malnutrition, haemochromatosis, autoimmune liver disease and/or chronic viral hepatitis were excluded. In addition, since the participants were general adult subjects underwent physical examinations, subjects with abnormal liver function were not included in this study. Finally, 8817 out of 10109 patients were included in this study.

### Data collection

A questionnaire was self-administered by all participants; the items included age, sex, ethnicity, medical history, family history, smoking history and alcohol abuse. Anthropometric data were measured using standard methods with the participants dressed in lightweight clothing and with bare feet [37]. The BMI was calculated as body weight divided by height squared (kg/m^2^) [37]. According to the categories recommended by the Chinese Ministry of Health, BMI < 18.5 refers to underweight, 18.5 < BMI < 23.9 refers to healthy weight, 24.0 < BMI < 27.9 refers to overweight, and BMI > 28.0 refers to obese. Blood pressure was measured using a mercury sphygmomanometer in a seated position after a 5-min rest and was recorded as the mean of two different measurements taken at 1-min intervals. A fasting blood sample was collected from each participant via the antecubital vein in the morning. Glucose; serum lipids including total cholesterol (TC), triglycerides (TG), low-density lipoprotein-C (LDL-C), high-density lipoprotein-C (HDL-C), apolipoprotein (apo)A, apoE, apoB and lipoprotein (a) Lp(a); indicators of liver function including alanine aminotransferase (ALT) and aspartate aminotransferase (AST); indicators of kidney function, including urea nitrogen, uric acid and creatinine were measured in the hospital laboratory according to routine procedures.

### Statistical analysis

The distribution tendency of BMI, age, systolic pressure, diastolic pressure, TC, TG, HDL-C, LDL-C, apoA, apoB, apoE, Lp(a), glucose, ALT, AST, urea nitrogen, uric acid and creatinine were examined using nonparametric tests. The correlation pattern between all variables and NAFLD was explored primarily using scatter and P-P diagrams. Pearson's correlation coefficients between each continuous variable were calculated prior to further statistical analysis. Collinearity diagnostics of all variables were also performed before further statistical analysis. After integrating the clinical significance, Pearson's correlation coefficients and collinearity diagnostics, TC, TG, HDL-C and LDL-C were selected to represent the serum lipid profile.

To identify the risk factor panels associated with NAFLD in subjects with different BMI values, the subjects were placed into underweight (BMI < 18.5), normal (18.5 < BMI < 23.9), overweight (24.0 < BMI < 27.9) and obese (BMI > 28.0) groups according to the definition recommended by the Chinese Ministry of Health. Binary logistic regression was performed in each BMI group, and similar selected variable sets were introduced into the starting model and then eliminated manually using the backward step-by-step approach, depending on the largest *p*-value. All items showing statistical significance at *p* < 0.05 were retained in the final equation. To evaluate the performance of each NAFLD prediction model, steady risk factors and integrated steady risk factors for males and females in the discrimination of NAFLD, area under the receiver operating characteristic (ROC) curve (AUC) analyses were also performed. All analyses were performed using SPSS for Windows (version 18).
